# The Practice Market

**Published:** 1911-04-01

**Authors:** 


### SPECIAL ARTICLES.

### THE PRACTICE MARKET.

### III.—TAKING A PARTNER.

In our last article we discussed in some detail the transfer of a general practice, strictly from the vendor's point of view, and incidentally we touched here and there upon the allied subject of taking in a partner. Of course many so-called "partnerships" of short duration are in reality merely extended forms of "introduction," with a view to the succession of the newcomer in a few months' time. In the present article we are not concerned with nominal partnerships of that description, but with the taking in of a *bonâ-fide* partner by an established practitioner for the benefit of his practice.

#### Hints to the Vendor.

Many of the principles which we have already laid down apply with equal force to the disposal of a share of a practice, and we do not intend to repeat those points which are obviously applicable to both cases. In future articles we shall deal very fully with the transfer of practices and partnerships from the purchaser's standpoint, but in this chapter, as in the last, our remarks are aimed exclusively at the vendor.

The replacement of an outgoing partner (who has possibly been the mainstay of the practice for many years), or the taking in of a partner for the first time in the history of the practice, is a very serious matter to everybody concerned, and especially to the remaining partner. The importance of finding a suitable man cannot be exaggerated. A thoroughly bad partner can well-nigh ruin a firm in a short time.

Every practitioner who, from whatever cause, proposes to take in a partner, has to face a big risk. Even when the incoming partner is personally known to the vendor and has perhaps been well received by his patients in the capacity of locum-tenens or assistant, there is still a risk. Good locums do not necessarily make good colleagues, and good assistants may "play the very devil" as partners.

#### Partners and Agents.

What we have previously said with regard to the various methods of disposing of a practice apply almost exactly to the taking in of a partner. The best partners are nearly always obtained privately, or semi-privately through friends on the staff of big teaching hospitals; but most medical men, seeking partners, are forced for one reason or another to enter their names sooner or later upon the books of an agent. If a partner is needed in a hurry, it is unlikely that a suitable man will be found at once through "a friend at court." The agent's commission is a heavy tax, but a good agent will do his utmost to suit the vendor, and his advice upon many matters of vital importance is often of the greatest value. Moreover, he usually has upon his books the names of many applicants for practices or partnerships, and among them there are often men of first-class attainments who are only prepared to look into exceptional openings. Almost every junior man, unless he has a practice waiting for him, finds his way into the office of one or more of the better-known agencies, since this step involves no expense to himself and may possibly lead to a sound investment.

#### The Partner's Wife.

When considering the claims of a prospective partner, the practitioner will naturally bear in mind the class and scope of his practice, and his own personal tastes and requirements. The incomer must satisfy him in various respects, such as age and physique, social status, manner and appearance, degrees and diplomas, and experience of hospital and private practice. The vendor should offer the hospitality of his house for a day or two to any likely candidate, and should discuss the matter with him with complete frankness. If the prospective partner is married or engaged (as is usually the case) it is an excellent plan for the vendor to interview not only the purchaser but also his wife or fiancée. A doctor's wife is either an invaluable asset to him or else she is very much the reverse. If possible the several partners' wives should agree, but this is most unlikely, and the great thing is that the newcomer's wife should not prove a drag upon the practice.

In arranging terms of partnership, the vendor should remember that he is taking a partner "for better, for worse." If he has secured a capable, energetic and loyal partner, the increase which follows this introduction of new blood into the practice will be well worth paying for by the transfer at a future date of a larger share of the receipts. It must be settled in the beginning whether the increased share shall be purchased by the newcomer on the basis upon which he entered the firm, or according to the receipts after he became a partner. In our opinion the vendor will do well not to stand out for the latter terms, which are often most inequitable to the incomer.

#### An Example.

A partner is generally taken in at one and a half to two and a half years' purchase of the share allotted to him, based as usual upon the average gross annual receipts of the practice for the three years preceding his entry. Thus if the average cash receipts are £1,500 and he buys a third share at two years' purchase, the vendor receives from him at the outset £1,000. These and many other points must be settled precisely and amicably by the principals, before the partnership deed is drawn up by legal hands and the practice enters upon its new phase.

(*To be continued.*)

